# Development of supercritical technology to obtain improved functional dietary fiber for the valorization of broccoli by‐product

**DOI:** 10.1002/jsfa.13990

**Published:** 2024-11-04

**Authors:** María Ángeles Rivas, María J. Benito, Alberto Martín, María de Guía Córdoba, Yesuneh Gizaw, Rocío Casquete

**Affiliations:** ^1^ School of Agricultural Engineering University of Extremadura Badajoz Spain; ^2^ University Institute of Agro‐Food Resources Research (INURA), Campus Universitario, University of Extremadura Badajoz Spain

**Keywords:** broccoli leaves, bioactive compounds, functional properties, supercritical fluid treatment (SFT), response surface methodology (RSM)

## Abstract

**Background:**

This research aimed to enhance the functional value of dietary fiber from broccoli leaves using supercritical fluid technology. By optimizing pressure, temperature, and time parameters through response surface methodology, the study sought to improve the bioactive properties of the fiber and develop a predictive model for its chemical composition and functional properties.

**Results:**

Structural analysis indicated that modified samples had a higher concentration of oligosaccharides than control samples did, with significant increases in galacturonic acid and neutral sugars after supercritical fluid technology treatment, highlighting enhanced pectin release due to cell wall degradation. Functional properties, such as water solubility, glucose absorption capacity, and antioxidant activity, improved significantly under optimized conditions (191 bar, 40 °C, 1 h). Multivariate analysis confirmed the effectiveness of supercritical fluid technology in enhancing the dietary fiber properties, achieving a global desirability value of 0.805.

**Conclusion:**

These results underscore the potential of supercritical technology for valorizing broccoli leaf by‐products, enhancing their health‐promoting characteristics and functional applications in the food industry. © 2024 The Author(s). *Journal of the Science of Food and Agriculture* published by John Wiley & Sons Ltd on behalf of Society of Chemical Industry.

## INTRODUCTION

Broccoli (*Brassica oleracea* L. var. *italica*) is a widely consumed food worldwide,^1^ with an increasing economic importance^2^, ^3^ owing to the health benefits because of its rich chemical composition.^4^, ^5^, ^6^ The edible part of broccoli has been the main focus of research, although it only constitutes approximately 10–15% of the plant weight.^7^ Therefore, it is necessary to investigate broccoli by‐products to find new alternatives and uses. Broccoli by‐products include non‐commercial leaves, stems, and inflorescences, and are widely recognized for their high content of dietary fiber and bioactive compounds.^8^ Consequently, they are valuable candidates for use as functional ingredients with health benefits.^9^, ^10^, ^11^ In particular, broccoli plant leaves are a significant source of vitamins, polyphenols, glucosinolates, and dietary fiber.^12^, ^13^, ^14^, ^15^, ^16^, ^17^

Dietary fiber is an essential component of a healthy diet and plays a critical role in the prevention of several chronic diseases, such as obesity, type 2 diabetes, and cardiovascular diseases.^18^, ^19^, ^20^, ^21^, ^22^, ^23^ In addition, several studies have suggested that dietary fiber may also play an important role in cancer prevention.^24^ Ren *et al*.^25^ characterized soluble dietary fiber from millet and reported its antitumor effect on colon cancer. In their study, they found that glucan inhibited cancer cell colony development and induced apoptosis. For this reason, its inclusion in the daily diet is recommended due to its great health benefits.

Bioactive activities of dietary fiber are closely related to its composition and solubility.^26^ It has been reported in several studies that soluble dietary fiber shows superior bioactive properties compared with insoluble dietary fiber.^27^ However, in plant by‐products, the presence of insoluble dietary fiber is predominant,^28^ which requires purification processes to improve its properties and release its potential. In recent research, several strategies have been explored to improve the functional properties of insoluble dietary fiber and adapt it to potential industrial applications in food. Rivas *et al*.^17^ demonstrated that enzymatic modification of dietary fiber increased the soluble dietary fiber content in broccoli by‐products, thus improving their oil‐holding capacity, solubility, and glucose absorption capacity; on the contrary, water‐holding capacity and swelling capacity were reduced. Xie *et al*.^29^ reported significant improvements in the antioxidant activity of dietary fiber from purple‐fleshed potatoes when these were treated with high pressures in their study. Purification of bioactive compounds yields a more concentrated and uniform product since it removes undesirable components, such as impurities and contaminants.^30^ In addition to these advantages, purified dietary fiber is usually more accessible and its functional properties can be more clearly focused.^31^ Thus, purification of dietary fiber offers an innovative approach that increases its potential applications both in the food industry and in promoting human health.

Supercritical technology, particularly the use of supercritical carbon dioxide (CO_2_) as a solvent, has emerged as a promising green and selective technique to purify bioactive compounds.^32^, ^33^ Response surface methodology (RSM) promises to be useful in this context, allowing adjustment of critical parameters, such as pressure, temperature, and time, to optimize the extraction and purification of specific compounds as demonstrated in different studies.^34^, ^35^, ^36^ RSM is a statistical and mathematical tool to explore and understand the complex interactions between multiple variables and their effects on a specific response.^37^, ^38^ RSM has been effective in the development, improvement, and optimization of complex processes, as recent studies support.^39^, ^40^ This methodology is highlighted by its economic efficiency, as it reduces significantly the amount of experiments needed while providing a large amount of data and information.^41^ Additionally, RSM allows one to analyze the simultaneous influence of multiple factors and anticipate the response of the system to new conditions, which facilitates determining the optimal conditions to obtain the desired response, as previous research has indicated. Imtiaz *et al*.^42^ reported that RSM was essential for optimizing the conditions of two extraction methods of bioactive compounds, microwave‐assisted extraction and ultrasound‐assisted extraction, designed to extract bioactive compounds from the leaves of *Thuja orientalis* tree. These models not only provided optimal conditions for the extraction of bioactive compounds and their functional activity, such as total phenolic content and antioxidant activity, but also allowed a deep understanding of the complex interactions between the factors involved.

Until now, research using this methodology to establish supercritical equipment conditions for dietary fiber modification has not been found. However, there are studies that have applied RSM to optimize dietary fiber extraction conditions by other methods. Behrouzian *et al*.^43^ used RSM to optimize the extraction of dietary fiber from coffee bean skin using alkaline hydrogen peroxide. In this work, the different factors such as temperature, hydrogen peroxide concentration, pH, and time were evaluated. The results demonstrated the effectiveness of this methodology to optimize the conditions for the extraction of dietary fiber.

Therefore, the aim of this research was to modify the dietary fiber obtained from broccoli leaves using supercritical technology. In this process, we optimized the pressure, temperature, and time parameters through RSM. Furthermore, we developed a model that predicts the chemical composition and functional properties of the modified dietary fiber under specific treatment conditions. To clarify the general objective of the research, the goal was to enhance the functional value of the dietary fiber derived from broccoli leaves through this innovative approach.

## MATERIALS AND METHODS

### Plant material

The broccoli by‐products used in this work were derived from cultivar ‘Parthenon’ in the region of Extremadura, Spain. The broccoli leaves’ by‐products were used. The samples were dried in a forced‐air oven at 45 °C for 24–48 h, ground with a mincer, packed in vacuum bags, and stored at room temperature until use. All the experiments were performed in triplicate.

### Total dietary fiber extraction

Total dietary fiber from broccoli leaves was extracted by the alcohol insoluble residue method, a suitable method for plant matrices with low starch content. Cell‐wall polysaccharides were extracted using the protocol described by Femenia *et al*.^44^ and modified by Rivas *et al*.^45^ Briefly, the dried samples were homogenized with 85% (v/v) ethanol and brought to boiling for 10 min, then filtered under vacuum through non‐cellulose filters (Whatman glass microfiber filters, 934‐AH™). This process was repeated twice more, the last time with absolute ethanol. The solid residue was washed with acetone and the excess solvent was removed after 24 h at a temperature of 23 ± 1 °C. This process was considered as a control.

### Dietary fiber treatment with supercritical fluids

Dietary fiber previously extracted from broccoli leaves was treated for its modification by supercritical fluid (SF) technology using CO_2_. A static unit in a Helix™ Speed SF System 96 (Applied Separations, Allentown PA, USA) was used for this process. A 20 g mass of dietary fiber was used, which was introduced into the 100 mL stainless steel equipment vessel. The solvent flow rate was set at 2 L min^−1^.

A Box–Behnken design (BBD) experiment with three factors and two blocks was applied to model the influence of the variables pressure, temperature, and time on the performance of dietary fiber modification (Table 1). The parameter ranges were defined after preliminary tests according to the conditions in previous studies by Rivas *et al*.^45^ After the process, the modified dietary fiber was stored under vacuum until analysis.

**Table 1:** Experimental runs of the Box–Behnken design per block for supercritical fluid treatment (SFT)

Pressure (bar)	Temperature (°C)	Time (h)
*Factorial points*		
225	55.0	3.00
300	47.5	1.00
225	55.0	1.00
300	55.0	2.00
150	47.5	3.00
300	40.0	2.00
150	55.0	2.00
225	40.0	3.00
300	47.5	3.00
150	40.0	2.00
150	47.5	1.00
225	40.0	1.00
Central points (SFT‐CP)		
225	47.5	2.00
225	47.5	2.00
225	47.5	2.00

### Characterization of dietary fiber

The characterization of dietary fiber was performed using the soluble part of the fiber. For this purpose, the samples were solubilized by mixing with water in a 1 : 10 ratio. The mixture was stirred for 30 min in a bath at a controlled temperature of 90 °C. Subsequently, the supernatant was precipitated by the addition of absolute ethanol at 60 °C. After centrifugation, the supernatant was removed and the soluble solid residue was dried at 45 °C.

#### Neutral sugars and galacturonic acid profile

The soluble residue was hydrolyzed using 12 mol L^−1^ sulfuric acid. This process was carried out for 3 h at room temperature, followed by an additional step at 100 °C for 1 h.

The neutral sugars and galacturonic acid profile of the soluble dietary fiber was determined by high‐performance liquid chromatography (HPLC) after hydrolysis using 12 mol L^−1^ sulfuric acid. This hydrolysis was carried out for 3 h at room temperature, followed by an additional step at 100 °C for 1 h.

HPLC analyses were performed using an Agilent LC 1260 Infinity II system (Waters, Milford, MA, USA) consisting of a separation module and a refractive index detector. A Rezex‐ROA column (7.8 mm ID × 150 mm; Phenomenex, Torrance, CA, USA) was used in an isocratic mode, with water as the mobile phase at a flow rate of 0.6 mL min^−1^. During elution, 10 μL of sample was injected, the column was maintained at a temperature of 80 °C, and the detector temperature was set at 40 °C.

#### Oligosaccharides

Soluble dietary fiber oligosaccharides in broccoli leaves were determined using HPLC. HPLC analyses were performed using an Agilent LC 1260 Infinity II system (Waters) consisting of a separation module and a refractive index detector. A Rezex‐RNO column (10 mm inner diameter × 200 mm; Phenomenex) was used in an isocratic mode, with water as the mobile phase at a flow rate of 0.6 mL min^−1^. During elution, 10 μL of sample was injected, the column was maintained at a temperature of 75 °C, and the detector temperature was set at 40 °C.

### Functional properties of dietary fiber

#### Water solubility

The water solubility (Ws) of the samples was determined following the method described by Wan *et al*.^46^ with slight modifications. A 0.5 g sample was weighed and mixed with 10 mL of distilled water. Then it was incubated at 90 °C for 30 min in a thermostatically controlled water bath, followed by centrifugation at 2000 × *g* for 15 min. The supernatant was collected, dried, and weighed. Ws was expressed as a percentage and was performed in triplicate.

#### Swelling, water retention capacity, and fat absorption capacity

Swelling (Sw), water retention capacity (WRC), and fat absorption capacity (FAC) of broccoli leaf dietary fiber were determined following the methodology described by Garau *et al*.^47^ For the determination of Sw, 0.1 g of fiber was mixed with 10 mL of distilled water in a graduated cylinder. After 24 h of incubation at room temperature (20–25 °C), the increase in volume was measured, and the results were expressed as milliliters of water per gram of fiber. For WRC, 0.2 g of fiber was hydrated in 10 mL of distilled water and left for 24 h at the same temperature. The next day, the sample was centrifuged at 2000 × *g* for 25 min. After centrifugation, the supernatant was decanted and the resulting solid residue was weighed. WRC was expressed as grams of water per gram of fiber. For FAC, the same procedure as for WRC was followed, but using 5 mL of sunflower oil instead of water. The results were expressed as grams of oil per gram of fiber.

#### Glucose absorption capacity (GAC)

The glucose absorption capacity (GAC) of dietary fiber was analyzed according to the method described by Niu *et al*.^48^ with some modifications. Briefly, 0.1 g of sample was mixed with 25 mL of a 50 mmol L^−1^ glucose solution. This was incubated at 37 °C for 6 h with constant shaking and then centrifuged at 4500 × *g* for 10 min. The glucose content of the supernatant was analyzed using HPLC equipment. GAC was expressed as milligrams glucose retained/milligram dietary fiber.

#### Antioxidant activity (non‐extractable polyphenol)

Non‐extractable polyphenols bound to dietary fiber from broccoli leaves were extracted according to the method described by Arranz *et al*.^49^ and determined using Folin–Ciocalteu reagent.^50^ The antioxidant capacity of the samples was evaluated by the 2,2‐diphenyl‐1‐picrylhydrogen oxide (DPPH) depletion method and the ability to scavenge the 2,2′‐azino‐bis(3‐ethylbenzothiazoline‐6‐sulfonic acid) (ABTS) radical.^51^, ^52^

### Statistical analysis

As shown in Table 1, a two‐block, three‐level, three‐factor BBD with 15 experimental runs per block (12 at factor points and three in the middle) combined with RSM was applied to determine the effects of supercritical fluid treatment (SFT) conditions on the composition and functional properties of dietary fiber modified from broccoli leaf. RSM was performed using StatGraphics Centurion XVI Version 8.0 software. The quadratic model was as follows:

$$Y=\beta_{0}+\beta_{1}X_{1}+\beta_{2}X_{2}+\beta_{3}X_{3}+\beta_{12}X_{1}X_{2}+\beta_{13}X_{1}X_{3}+\beta_{23}X_{2}X_{3}\;+\beta_{11}X_{1}^{2}+\beta_{22}X_{2}^{2}+\beta_{33}X_{3}^{2}+\varepsilon$$

where *Y* is the response variable predicted by the model, *β*_0_ is an offset value, *β*_1_, *β*_2_, and *β*_3_ are the regression coefficients for the main (linear) terms, *β*_11_, *β*_22_, and *β*_33_ are quadratic effects, *β*_12_, *β*_13_, and *β*_23_ are interaction effects, *X*_1_, *X*_2_, and *X*_3_ are the independent variables, and *ε* is the experimental error. For each experimental factor, the software generated an analysis of variance (ANOVA), establishing statistical significance at the 95% confidence level. The same statistical program was also used to obtain response surface graphs and the optimum level for each variable analyzed. An optimization of multiple responses using Derringer's desirability function was achieved with the variables that showed a goodness of fit to the models.

One‐way ANOVA was performed for the comparison of control and SFT central point (SFT‐CP). In addition, to reduce the dimensionality of data sets and to improve the global visualization, principal component analysis (PCA) was achieved with the values of the components and functional properties of dietary fiber treated with supercritical fluids (Supporting Information).

## RESULTS AND DISCUSSION

### Effect of SFT conditions on the composition and structure of broccoli leaf dietary fiber

#### Dietary fiber structure

According to Naveed *et al*.,^53^ oligomers consisting of 2 to 20 monosaccharide units are known as oligosaccharides. The detected peak has a degree of polymerization (DP) between 7 and 12, suggesting that the samples analyzed can be classified as oligosaccharides. Table 2 shows the results of the structural analysis of broccoli leaf dietary fiber. The modified samples and the control sample showed the same oligosaccharide or group of oligosaccharides but at different concentrations. The control sample showed the lowest value (592.94 g kg^−1^) in the DP concentration (7–12), compared with the SFT‐CP samples (899.49 g kg^−1^) and even the lowest value (769.35 g kg^−1^) when the SFT conditions included in the BBD were applied (Table 2).

**Table 2:** Comparison of control and supercritical fluid treatment‐central points (SFT‐CP) samples and descriptive statistics of runs included in Box–Behnken design (BBD) for oligosaccharide degree of polymerization (DP) and soluble fiber constituents

	One‐way analysis of variance (mean ± SD)			BBD runs (descriptive statistics)		
	Control	SFT‐CP	*P* *	Mean ± SD	Max.	Min.
DP (7–12)	592.94 ± 30.87	899.49 ± 53.33	0.000	947.46 ± 127.26	998.91	769.35
Soluble fiber constituents						
Galacturonic acid	98.54 ± 0.02	98.31 ± 0.09	0.020	98.46 ± 0.34	98.91	97.77
Glucose	0.73 ± 0.01	0.86 ± 0.05	0.027	0.62 ± 0.11	0.87	0.44
Xylose	0.16 ± 0.05	0.03 ± 0.03	0.008	0.04 ± 0.04	0.16	0.01
Arabinose	0.00 ± 0.00	0.00 ± 0.00	—	0.24 ± 0.17	0.42	0.00
Fucose	0.56 ± 0.02	0.79 ± 0.04	0.001	0.62 ± 0.16	0.89	0.38

*Note*: DP (7–12): oligosaccharide DP between 7 and 12. DP (7–12) (mg g^−1^ fiber dietary); fiber constituents (p/p%). Values are represented as a mean plus/minus standard deviation (SD; *n* = 3).

* *P*‐values lower than 0.05 are statistically significant.

**Figure 1:**
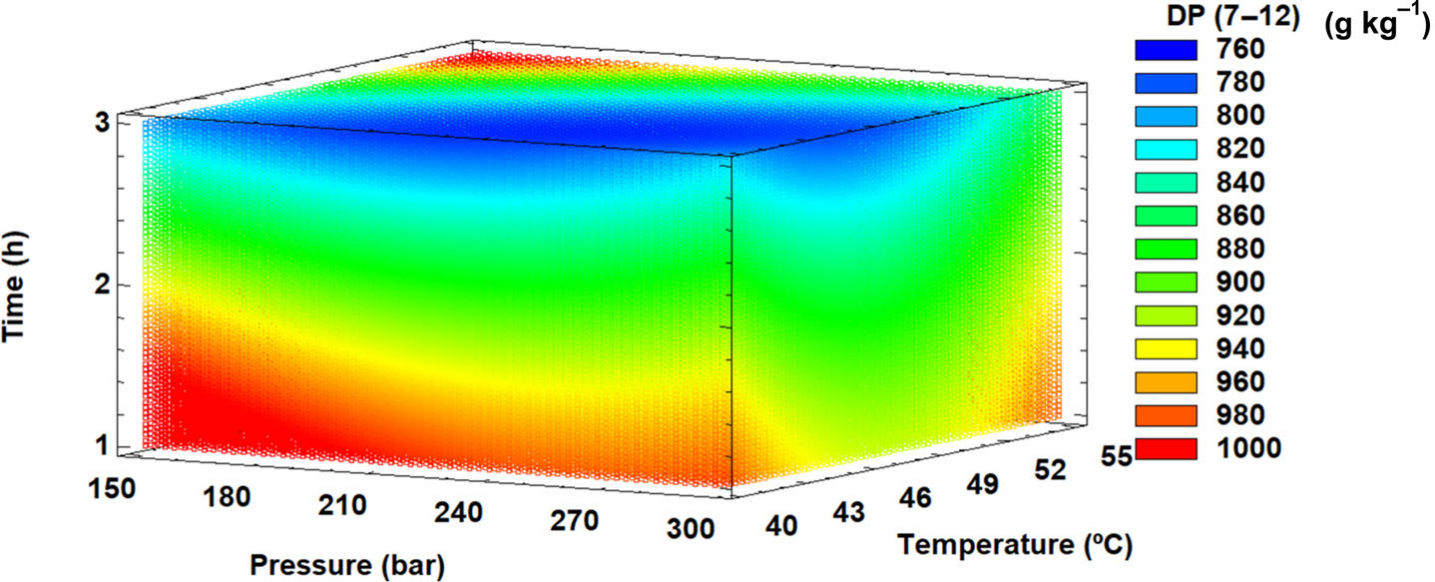
Response surface mesh for oligosaccharide degree of polymerization (DP) between 7 and 12 with respect to supercritical fluid treatment conditions.

Table 3 shows the results of ANOVA for the quadratic response surface model and optimal supercritical fluid conditions for each parameter of treated dietary fiber. The effects of SFT conditions on DP concentration (7–12) were adjusted to the designed quadratic model with an *R* ^2^ value of 0.70 (Table 3). Significant linear effects were observed with temperature, pressure, and time, as well as a quadratic effect with temperature. In addition, a significant interaction between pressure and temperature was identified. Figure 1 shows that, at lower pressures and shorter times, the DP values increased but decreased with lower temperatures, with the optimal conditions being for maximized DP concentration (7–12) at 55 °C, 150 bar, and 1 h treatment. As shown in Table 3, this optimal condition achieved values near to 100% DP (7–12), so it could be affirmed that the SFT with the appropriate conditions was able to modify the dietary fiber efficiently and produce a dietary fiber concentrate composed only of oligosaccharides. The results suggest that the treatment of the dietary fiber matrix with appropriate temperatures and pressures during the supercritical process causes the breakdown of the polysaccharides’ molecular chain and the reduction of the molecular weight.^54^, ^55^ However, further studies are needed to ensure the economic viability of producing large quantities of these oligosaccharides.

**Table 3:** Analysis of variance (ANOVA) of quadratic response surface model and optimal supercritical fluid treatment (SFT) for oligosaccharide degree of polymerization (DP) and soluble fiber constituents

		Soluble fiber constituents				
Source	DP (7–12)	Galacturonic acid	Glucose	Xylose	Arabinose	Fucose
ANOVA (*P*‐values)						
A: Pressure	**0.000** *	0.173	0.667	0.662	0.810	**0.024**
B: Temperature	**0.001**	**0.000**	**0.000**	0.950	0.238	**0.000**
C: Time	**0.000**	**0.000**	**0.000**	0.387	**0.000**	**0.006**
AA	0.223	0.246	**0.000**	0.302	**0.000**	**0.044**
AB	**0.029**	0.329	0.099	0.482	0.3349	0.203
AC	0.364	**0.011**	0.154	0.105	0.641	**0.002**
BB	**0.023**	0.093	**0.000**	0.517	**0.000**	**0.000**
BC	0.541	**0.000**	0.807	0.430	**0.000**	0.802
CC	0.530	**0.017**	**0.003**	0.091	0.430	**0.003**
*ANOVA (R* ^2^ *statistics*)						
*R* ^2^	0.80	0.91	0.89	0.34	0.87	0.88
*R* ^2^ (adjusted by DF)	0.70	0.86	0.83	0.02	0.80	0.81
*Optimal SFT conditions*						
Pressure (bar)	150	300	223	—	150	233
Temperature (°C)	54.8	55.0	44.4	—	40.0	42.7
Time (h)	1.00	1.00	2.40	—	3.00	2.30
Estimated value	999	98.9	0.89	—	0.69	0.85

*Note*: DP (7–12): oligosaccharide degree of polymerization between 7 and 12. DP (7–12) (mg g^−1^ fiber dietary); fiber constituents (p/p%); DF, degrees of freedom. Values are represented as a mean plus/minus standard deviation (*n* = 3).

* *P*‐values lower than 0.05 (in bold) are statistically significant.

Dietary fiber modification by supercritical fluids to obtain oligosaccharides is a topic for research. However, there are a few studies investigating the use of supercritical fluids in the extraction of polysaccharides. Gong *et al*.^56^ used supercritical fluid extraction (SFE‐CO_2_) with ethanol as co‐solvent to obtain a polysaccharide from ginkgo leaves, and the results showed that the polysaccharides obtained had lower molecular weight than the polysaccharides derived from ginkgo leaves obtained by hot water extraction. In a more recent study, Manthei *et al*.^57^ investigated the use of apple bagasse and orange peel by subjecting them to high‐pressure homogenization, enzymatic hydrolysis, and a combination of both to evaluate the production of oligosaccharides. The results showed that pretreatment of the substrates with high‐pressure homogenization enhanced the release of pectic oligosaccharides in orange peel, reaching a content of 44.51 g/100 g peel.

#### Dietary fiber composition

**Figure 2:**
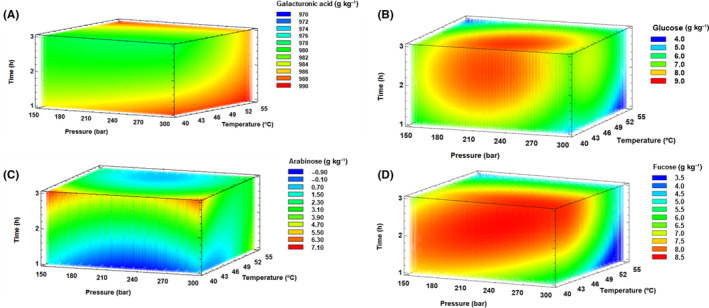
Response surface mesh for galacturonic acid (A), glucose (B), arabinose (C), and fucose (D) with respect to supercritical fluid treatment conditions.

Table 2 shows that the results of galacturonic acid showed differences between the control and SFT‐CP, showing concentrations of 985.4 g kg^−1^ and 983.1 g kg^−1^ respectively. However, analysis of the experimental runs using the response surface design (BBD) shows a maximum value of 989.1 g kg^−1^. Owing to the high content of galacturonic acid, it can be stated that the pectin concentration is high. The typical structure of pectin is composed of a backbone consisting of galacturonic acid linked by *α*‐d‐1,4‐glycosidic bonds, with the backbone connected to side chains containing neutral sugars through various glycosidic bonds.^58^, ^59^ This result seems to indicate that the dietary fiber analyzed is composed almost entirely of homogalacturonan domain as a very high percentage of its composition is galacturonic acid, and to a lesser extent of ramnogalacturonan domains I and II, which have complex side chains containing neutral monosaccharides such as arabinose, galactose, and others.^60^ Furthermore, galacturonic acid content adjusted adequately to the designed quadratic model with a high *R* ^2^ coefficient value of 0.91 (Table 3). As the pressure and temperature increased, higher values of galacturonic acid were purified, obtaining the maximum estimated values (989 g kg^−1^) at 300 bar pressure, 55 °C temperature, and 1 h extraction time (Fig. 2(A) and Table 3).

The results of the neutral sugars as constituents of broccoli leaf dietary fiber are also presented in Table 2. Glucose and fucose were the major neutral sugars, both showing lower values in the control samples (7.3 g kg^−1^ and 5.6 g kg^−1^ respectively) compared with the SFT‐CP samples (8.6 g kg^−1^ and 7.9 g kg^−1^ respectively) (*P* < 0.05). On the other hand, xylose had a significantly lower value in the SFT‐CP samples compared with the control sample. Finally, arabinose was not found in either the control or the SFT‐CP samples. However, it should be noted that the maximum values of all monosaccharides were higher when the SFT conditions included in the BBD were applied (Table 2). The concentration of all monosaccharides adjusted the designed quadratic model with *R* ^2^ values higher than 0.8 in all cases except for xylose (Table 3). It is also worth mentioning that all monosaccharides presented maximum conditions in terms of pressure, although all of them coincided in similar temperatures between 44 and 40 °C and treatment times between 2 and 3 h. Figure 2(B),(D) shows that glucose and fucose values decreased at higher temperatures, and in the case of arabinose (Fig. 2(C)) the values increased at the extreme points of pressure during long times and lower temperatures.

Neutral sugars, together with galacturonic acid, show an increase after SFE treatment compared with the control (Table 2). This increase could be attributed to a higher pectin release as a consequence of cell wall degradation accompanied by the breaking of glycosidic bonds, which has been observed by other researchers when treating and modifying dietary fiber fractions by different methods.^61^, ^62^

Although there are no specific studies on the dietary fiber purification using supercritical fluids, several investigations have explored the effects of temperature and pressure on dietary fiber pectin as well as galacturonic acid content. In addition, some studies support the use of supercritical CO_2_ to improve dietary fiber pectin. In a previous study, the impact of temperature, pressure, and extraction time on pectin from pomegranate peel dietary fiber treated with supercritical fluids was evaluated. Specific conditions, such as a temperature of 45 °C, a pressure of 300 bar, and an extraction time of 2 h, were found to maximize the galacturonic acid content.^44^ The optimal time value is higher than the one achieved in this work (Table 3); however, the plant material is different, and in this work the supercritical treatment was applied directly on the dietary fiber fraction, which would leave the pectin present and the rest of its components more exposed. Pedraza‐Guevara *et al*.^63^ focused their research on the development of environmentally friendly and sustainable processes using compressed fluids to obtain high‐quality pectin from green papaya flour. Their research highlighted that the introduction of CO_2_ into the matrix caused the breaking of hydrogen bonds, increasing the porosity of the cell membrane and facilitating the recovery of pectin, without affecting the monosaccharides and promoting a high content of galacturonic acid. In another study, Xie *et al*.^64^ explored the effects of high‐pressure processing on waste potato peel pectins, finding an increase in galacturonic acid content and a decrease in the degree of esterification. This work underlines the ability of high pressure to modify pectin characteristics. Benvenutti *et al*.^65^ studied the recovery of pectin from jaboticaba by‐product by subcritical water extraction modified by deep eutectic solvent. They identified that the maximum pectin yield was reached at 122 °C; higher temperatures caused a decrease in yield, possibly due to damage to the pectin structure.

### Effect of SFT conditions on the functional properties of broccoli leaf dietary fiber

Table 4 presents the descriptive statistics of the functional properties of the treatment of dietary fiber under the SFT conditions set out in the BBD, and the differences found between the control and SFT‐CP samples. In general, SFT‐CP samples showed significantly higher values (*P* < 0.05) than the control samples for all functional properties except FAC.

**Table 4:** Comparison of control and supercritical fluid treatment‐central points (SFT‐CP) samples and descriptive statistics of runs included in Box–Behnken design (BBD) for all properties studied

	One‐way analysis of variance (mean ± SD)			BBD runs (descriptive statistics)		
	Control	SFT‐CP	*P* *	Mean ± SD	Max.	Min.
Ws	11.98 ± 0.94	15.00 ± 0.94	0.001	14.34 ± 2.35	17.98	9.02
Sw	8.84 ± 0.62	10.49 ± 0.64	0.003	9.14 ± 1.78	12.38	5.82
WRC	9.84 ± 0.62	11.49 ± 0.64	0.003	10.14 ± 1.78	13.38	6.82
FAC	7.36 ± 0.53	7.90 ± 0.73	0.267	8.03 ± 1.33	10.95	5.63
GAC	0.72 ± 0.01	0.92 ± 0.12	0.023	0.87 ± 0.22	1.25	0.55
FNE	215.28 ± 15.91	355.95 ± 26.68	0.000	403.77 ± 64.41	509.19	266.84
DPPH	84.57 ± 3.14	108.07 ± 15.66	0.031	137.65 ± 17.72	176.33	100.65
ABTS	399.40 ± 8.34	459.84 ± 36.99	0.021	488.39 ± 27.28	545.15	440.47

*Note*: Values are represented as a mean plus/minus standard deviation (SD; *n* = 3).

Abbreviations: Ws, solubility (%); Sw, swelling (mL g^−1^); WRC, water retention capacity (g g^−1^); FAC, fat adsorption capacity (g g^−1^); GAC, glucose absorption capacity (g g^−1^); FNE, total non‐extractable phenolic compounds (mg gallic acid equivalent/100 g); DPPH, 2,2‐diphenyl‐1‐picrylhydrogen oxide (antioxidant activity of extract; mg Trolox/100 g); ABTS, 2,2′‐azino‐bis(3‐ethylbenzothiazoline‐6‐sulfonic acid) (antioxidant activity of extract; mg Trolox/100 g).

* *P*‐values lower than 0.05 are statistically significant.

#### Effect of SFT conditions on the Ws of dietary fiber

The Ws values ranged from 17.98 to 9.02% when the SFT conditions included in the BBD were applied (Table 4). Significant effects (*P* < 0.05) were observed for pressure (quadratic), temperature (linear), and the interaction of pressure and time on Ws (Table 5).

**Table 5:** Analysis of variance (ANOVA) of quadratic response surface model and optimal supercritical fluid treatment (SFT) conditions for the functional properties studied

Source	Ws	Sw	WRC	FAC	GAC	FNE	DPPH	ABTS
*ANOVA* (*P‐values*)								
A: Pressure	0.135	0.352	0.352	**0.045**	**0.000**	0.750	0.199	0.759
B: Temperature	**0.022**	**0.001**	**0.001**	0.393	**0.030**	**0.000**	**0.000**	**0.041**
C: Time	0.131	0.355	0.355	0.836	0.073	0.075	**0.001**	0.783
AA	**0.013**	0.945	0.945	0.420	**0.000**	0.376	0.053	0.603
AB	0.060	0.213	0.213	**0.000**	**0.048**	**0.027**	0.354	0.753
AC	**0.000**	0.554	0.554	0.233	0.825	**0.002**	**0.000**	0.092
BB	0.168	**0.000**	**0.000**	0.568	0.122	0.398	**0.000**	0.072
BC	0.062	**0.000**	**0.000**	**0.023**	**0.000**	**0.004**	**0.008**	0.493
CC	0.382	0.291	0.291	**0.049**	**0.000**	**0.000**	**0.000**	**0.016**
*ANOVA (R* ^*2*^ *statistics*)								
*R* ^2^	0.59	0.66	0.66	0.50	0.75	0.87	0.83	0.35
*R* ^2^ (adjusted by DF)	0.46	0.55	0.55	0.34	0.67	0.83	0.78	0.14
*Optimal SFT conditions*								
Pressure (bar)	—	150	150	—	191	150	150	—
Temperature (°C)	—	40.9	40.9	—	40.0	40.0	40.0	—
Time (h)	—	1.00	1.00	—	1.00	3.00	3.00	—
Estimated value	—	12.0	13.0	—	1.20	541	188	—

*Note: P*‐values lower than 0.05 are statistically significant (bold font).

Abbreviations: Ws, solubility (%); Sw, swelling (mL g^−1^); WRC, water retention capacity (g g^−1^); DF, degrees of freedom; FAC, fat adsorption capacity (g g^−1^); GAC, glucose absorption capacity (g g^−1^); FNE, total non‐extractable phenolic compounds (mg gallic acid equivalent/100 g); DPPH, 2,2‐diphenyl‐1‐picrylhydrogen oxide (antioxidant activity of extract; mg Trolox/100 g); ABTS, 2,2′‐azino‐bis(3‐ethylbenzothiazoline‐6‐sulfonic acid) (antioxidant activity of extract; mg Trolox/100 g).

With regard to dietary fiber modification, the results obtained indicate a linear increase in fiber solubility with increasing temperature, as revealed by several studies. Ullah *et al*.^66^ examined the properties of insoluble dietary fiber from okara and showed that the solubility of the insoluble dietary fiber increased steadily with increasing pretreatment temperature. On the other hand, the influence of pressure on solubility is more complex and less predictable, as was highlighted in the study of Ouyang *et al*.^67^ on grapefruit dietary fiber. Ultra‐high pressures above 500 MPa resulted in total degradation of dietary fiber, whereras pressures between 100 and 400 MPa increased solubility by converting insoluble fractions into soluble ones. In addition, previous studies, such as the one conducted by Xie *et al*.^29^ indicated that high pressure can convert insoluble cellulose and hemicellulose fractions into soluble ones, thus improving the solubility of dietary fiber.

#### Effect of SFE conditions on the functional properties of dietary fiber

**Figure 3:**
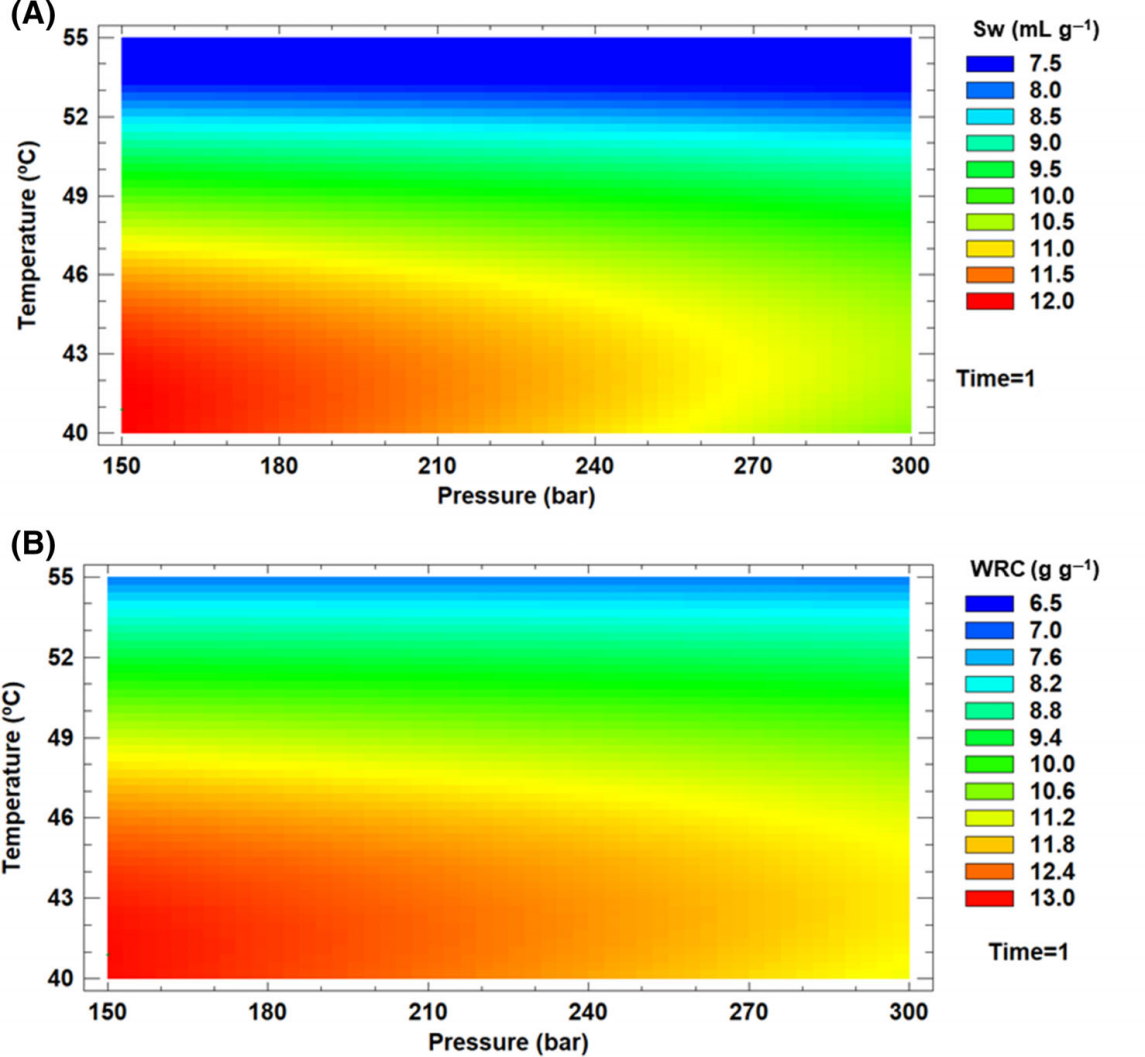
Contour plots for swelling capacity (Sw) with respect to temperature and pressure (A) and water retention capacity (WRC) with respect to temperature and pressure (B).

Sw, WRC, and FAC values of dietary fiber treated with SF from broccoli leaves are shown in Table 4. The maximum values found in the BBD experimental runs (Table 4) show that the efficiency of SFT under optimal conditions was clearly higher than that of the control samples for all technological properties. Sw and WRC did fit the model with an *R* ^2^ coefficient value of 0.55 in both cases. For both properties, the SFE conditions that showed significant effects were temperature (linear and quadratic effect) and the interaction between temperature and time (Table 5). The maximum values of Sw and WRC coincided with low temperatures (40.0 °C) and pressures (150 bar) and shorter purification times (1 h) (Fig. 3). In the case of FAC, the variability of the parameter was mainly associated with the interaction between pressure and temperature factors (Table 5).

The results suggested that the application of temperature and pressure by supercritical fluid improved these properties of the dietary fiber. However, the results also showed that too high temperatures and pressures degrade the dietary fiber, resulting in lower Sw and WRC (Fig. 3). The results obtained from different research support our findings and reveal the relevance of temperature and pressure in the modification of dietary fiber to improve its properties. Wang *et al*. showed that modifications on dietary fiber from lychee pomace by techniques such as high‐pressure homogenization and high hydrostatic pressure created more porous structures, improving its WRC, Sw, and FAC.^68^ Ouyang *et al*.^67^ also confirmed that ultra‐high pressure treatment significantly increased WRC and FAC of grapefruit dietary fiber, although excessive pressure damaged the internal structure, reducing these properties. On the other hand, Dong *et al*.^69^ used a combination of ultrasound and high‐temperature cooking to improve these properties of bamboo dietary fiber, increasing its WRC and FAC due to smaller particle size and looser structure. Similarly, Su *et al*.^70^ applied a lactic acid assisted subcritical water treatment to dietary fiber from beer bagasse, where high temperature (180 °C) during the process resulted in a more porous structure and smaller particles, significantly improving the WRC and FAC of dietary fiber. These studies underline the positive influence of temperature and pressure conditions on dietary fiber properties, showing how appropriate modification can lead to significant improvements in its functionality, which is crucial for its application in food products.

#### Effect of SFT conditions on GAC of dietary fiber

**Figure 4:**
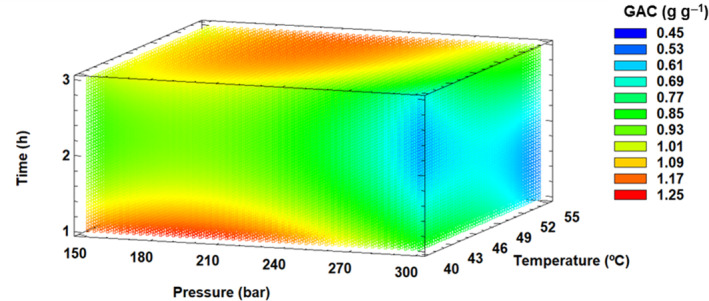
Response surface mesh for glucose absorption capacity (GAC) with respect to supercritical fluid treatment conditions.

Table 4 presents the descriptive statistics of GAC under the SFT conditions set out in the BBD, and the differences found between control and SFT‐CP samples. The SFE‐CP samples showed higher values (0.92 g g^−1^) than the control samples when GAC was measured (*P* < 0.05). GAC values ranged from 1.25 to 0.55 g g^−1^ when applying the SFT conditions included in the BBD, showing lower values in the control than in the SFT‐CP samples (Table 4). This parameter adjusted adequately to the designed quadratic model with an *R* ^2^ coefficient value of 0.67 (Table 5). In addition, Fig. 4 and Supporting Information Fig. S1 show that the GAC values increase at pressures between 150 and 240 bar at short times and low temperatures, estimating the maximum values (1.22 g g^−1^) at 191 bar pressure, 40 °C temperature, and 1 h (Table 5).

The GAC values obtained for the dietary fiber of the control sample are similar to those of previously published study, although they are also higher than those published by other workers.^45^, ^71^ As for the modification and improvement after treatment with supercritical fluid, the effects of time and pressure are evident. The differences can be attributed to the change in the structure of the dietary fiber after temperature and pressure conditions impact on the matrix, which could suggest an increase in the specific surface area that would allow a higher glucose absorption capacity.^72^

#### Effect of SFT conditions on phenol non‐extractable content and antioxidant capacity of dietary fiber

Table 4 presents the descriptive statistics of the phenol non‐extractable (FNE) content and antioxidant activity of the treated dietary fiber under the SFT conditions established in the BBD, and the differences found between the control samples and the supercritical center point conditions (SFT‐CP). The SFT‐CP samples showed higher values (*P* < 0.05) with respect to the control in both FNE content and antioxidant activity. In the BBD experimental runs (Table 4), it was observed that the maximum values of FNE (509.19 mg gallic acid equivalent (GAE)/100 g) and antioxidant activity measured by the DPPH (176.33 mg Trolox/100 g) and ABTS (545.15 mg Trolox/100 g) indicated a clear increase with SFT compared with control samples (251.25 mg GAE/100 g, 84.57 mg Trolox/100 g, and 399.40 mg Trolox/100 g respectively). Both FNE content and antioxidant activity by the DPPH method showed high *R* ^2^ coefficients of 0.83 and 0.78 respectively.

**Figure 5:**
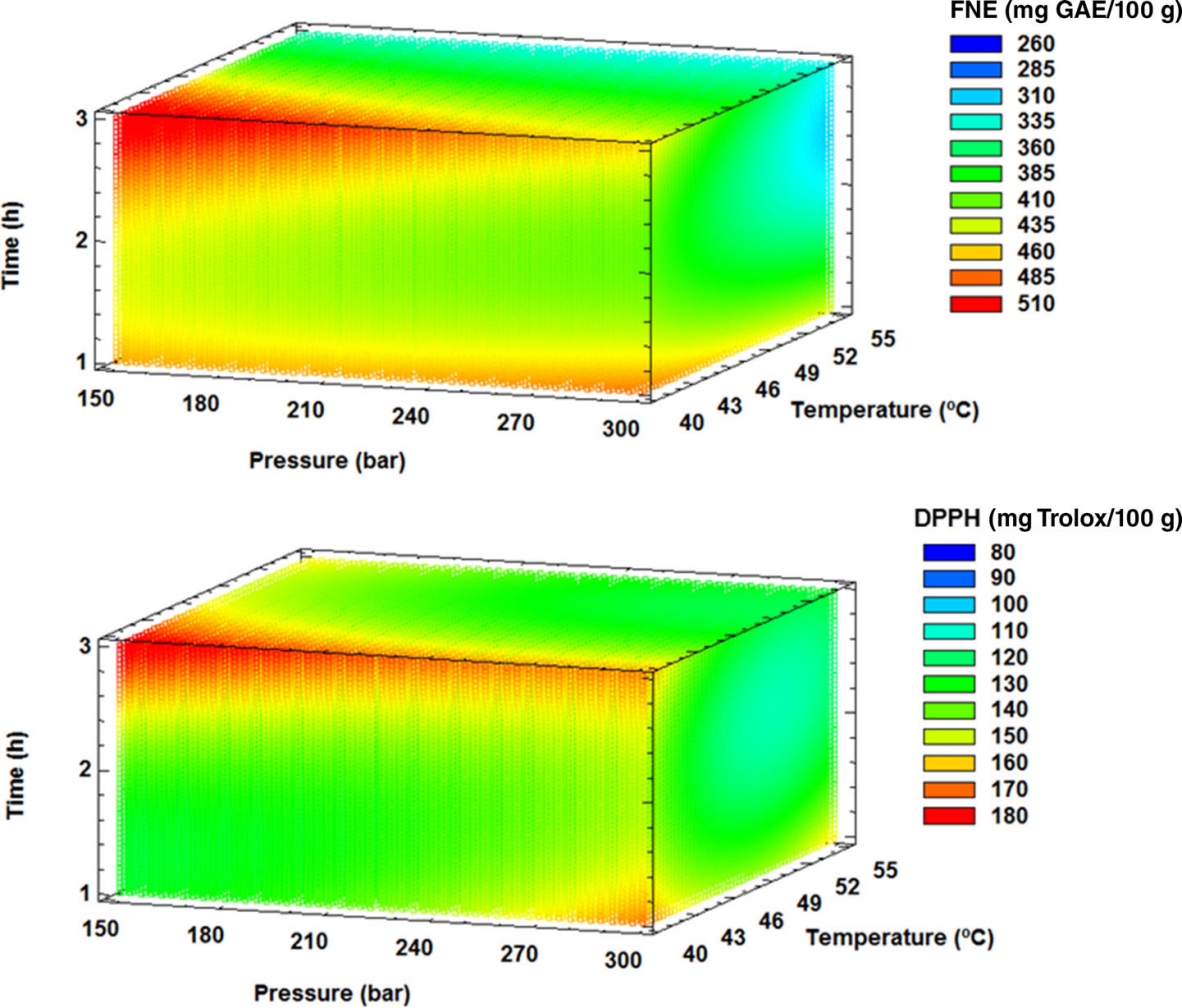
Response surface mesh for total non‐extractable phenolic compounds (FNE) (A) and antioxidant activity of extract (DPPH) (B) with respect to supercritical fluid treatment conditions.

Figure 5 shows that, under the conditions of low pressure (150 bar), low temperature (40 °C), and extended times (3 h), the FNE content and antioxidant activity (DPPH) approached a maximum estimated value of 541.62 mg GAE/100 g and 188.63 mg Trolox/100 g of dietary fiber respectively (Table 5). These results highlight the positive influence of these factors on FNE content and antioxidant activity and justify their use to purify and improve dietary fiber from broccoli leaves (Supporting Information Fig. S1).

In previous research, several improvement strategies, including supercritical fluid optimization, were employed to study the effect of temperature, pressure, and time parameters on dietary fiber in broccoli by‐products.^23^ Two specific supercritical conditions, 300 bar at 55 °C and 150 bar at 45 °C, both for 2 h, were explored to assess their impact on the functional properties of dietary fiber, including FNE content and antioxidant activity. The 300 bar and 55 °C condition was able to improve the levels of FNE content and antioxidant activity, as measured by the DPPH method, compared with the control group. It is important to mention that the pressure and temperature conditions in these previous studies exceed the optimum conditions obtained in the present work. However, these differences could be attributed to the treatment time. In another previous study, pomegranate peel, after supercritical extraction of phenolic compounds, was investigated, and it was determined that 3.8 h at a temperature of 55 °C and a pressure of 250 bar was needed to reach the maximum antioxidant activity in the dietary fiber extracted from the residue resulting from supercritical extraction.^45^ These findings underline the relevance of extraction time, as well as temperature and pressure, in improving the functional properties of dietary fiber in plant by‐products.

### Multivariate analysis of parameters related to SFT extracts and residual dietary fiber under different SFT conditions

**Figure 6:**
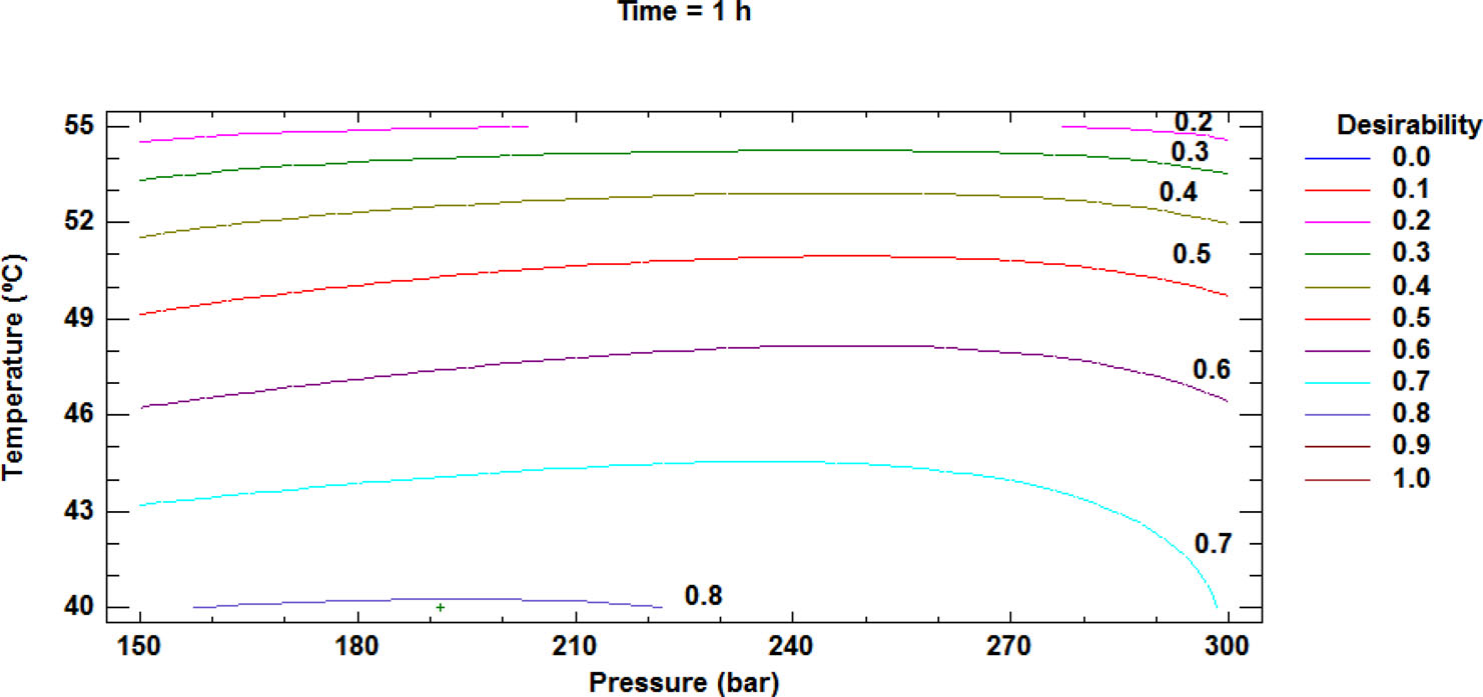
Contours of estimated response surface of multiple response optimizations for maximum values of swelling, water retention capacity, glucose absorption capacity, total non‐extractable phenolic compounds, and antioxidant activity of extract.

Based on the goodness‐of‐fit models, the functional properties Sw, WRC, GAC, total FNE, and antioxidant activity of extract (DPPH) were used for overall optimization of the SFT conditions (Table 5). Figure 6 shows the Derringer desirability function methodology for estimating optimal SFT conditions (191 bar pressure, 40 °C temperature, and 1 h time), with maximum values of Sw, WRC, GAC, FNE, and DPPH (11.53 mL g^−1^, 12.53 g g^−1^, 1.22, 467.60 mg GAE/100 g, and 134.55 mg Trolox/100 g respectively) and a global desirability value for this solution of 0.805.

## CONCLUSIONS

The results of this study indicate that the SFT conditions used for the treatment of dietary fiber obtained from broccoli leaves affect the composition and functional properties of the dietary fiber obtained. The use of RSM for the optimization of the supercritical fluid conditions resulted in the selection of acceptable experimental conditions to obtain dietary fiber with optimal composition and properties. It is important to note that the conditions for optimizing dietary fiber were not the same when focusing on achieving an optimal polymerization degree *versus* enhancing specific techno‐functional properties. This distinction highlights the versatility of the process. Supercritical technology emerges as a promising approach for revalorizing broccoli leaves, optimizing their properties for health benefits and industrial applications. However, the research also presents certain limitations, particularly regarding its large‐scale application. Further studies are needed to evaluate the feasibility and practical implications of implementing this technology on an industrial scale.

## CONFLICT OF INTEREST

The authors declare that they have no known competing financial interests or personal relationships that could have appeared to influence the work reported in this paper.

## Supporting information


**Figure S1.** Loading plot (A) and score plot (B) after principal component analysis of components and functional properties of dietary fiber treated with supercritical fluids in the planes defined by the two first principal components (PC_1 and PC_2). Ws, solubility; Sw, swelling; WRC, water retention capacity; FAC, fat adsorption capacity; GAC, glucose absorption capacity; FNE, total non‐extractable phenolic compounds; DPPH and ABTS, antioxidant activity of extract; Oligos, oligosaccharide degree of polymerization between 7 and 12; Agal, Galacturonic acid; Glu, Glucose; Xyl, Xylose; Ara, Arabinose; Fuc, Fucose.

## Data Availability

The data that support the findings of this study are available on request from the corresponding author. The data are not publicly available due to privacy or ethical restrictions.
