# Supplementary material for: Development of supercritical technology to obtain improved functional dietary fiber for the valorization of broccoli by‐product
Source: J Sci Food Agric. 2024 Nov 4;105(4):2203–14. doi: 10.1002/jsfa.13990 (PMC11824917; doi:10.1002/jsfa.13990)
Supplement: Supplementary file 1 — Figure S1. Loading plot (A) and score plot (B) after principal component analysis of components and functional properties of dietary fiber treated with supercritical fluids in the planes defined by the two first principal components (PC_1 and PC_2). Ws, solubility; Sw, swelling; WRC, water retention capacity; FAC, fat adsorption capacity; GAC, glucose absorption capacity; FNE, total non‐extractable phenolic compounds; DPPH and ABTS, antioxidant activity of extract; Oligos, oligosaccharide degree of polymerization between 7 and 12; Agal, Galacturonic acid; Glu, Glucose; Xyl, Xylose; Ara, Arabinose; Fuc, Fucose. [file JSFA-105-2203-s001.docx]

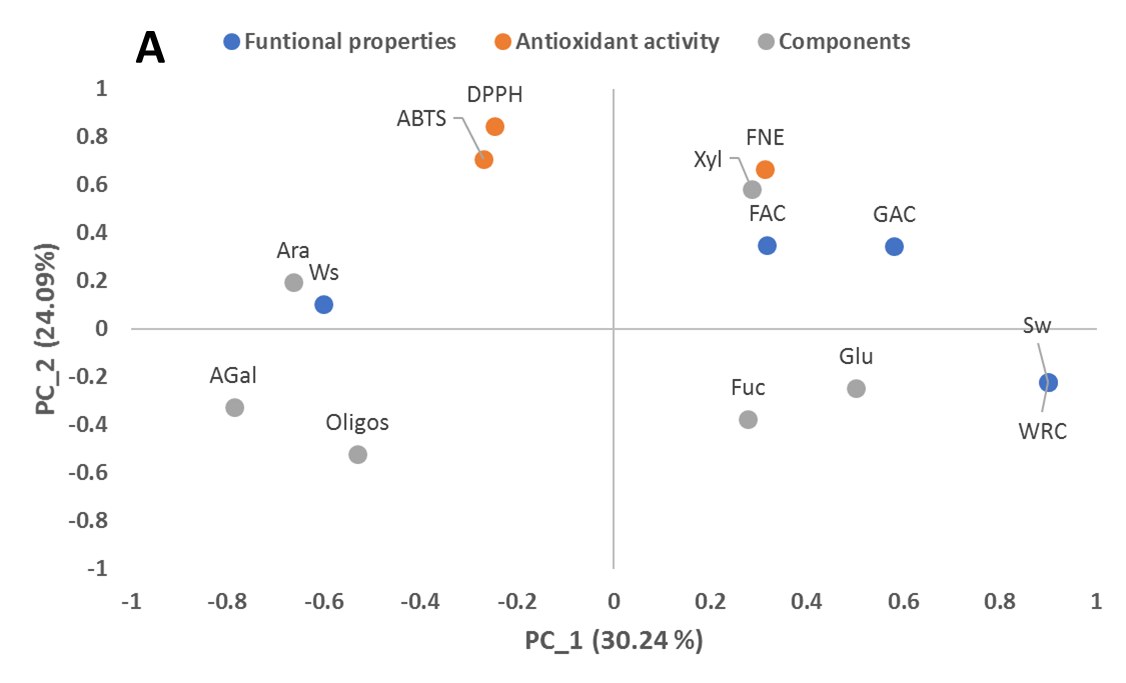


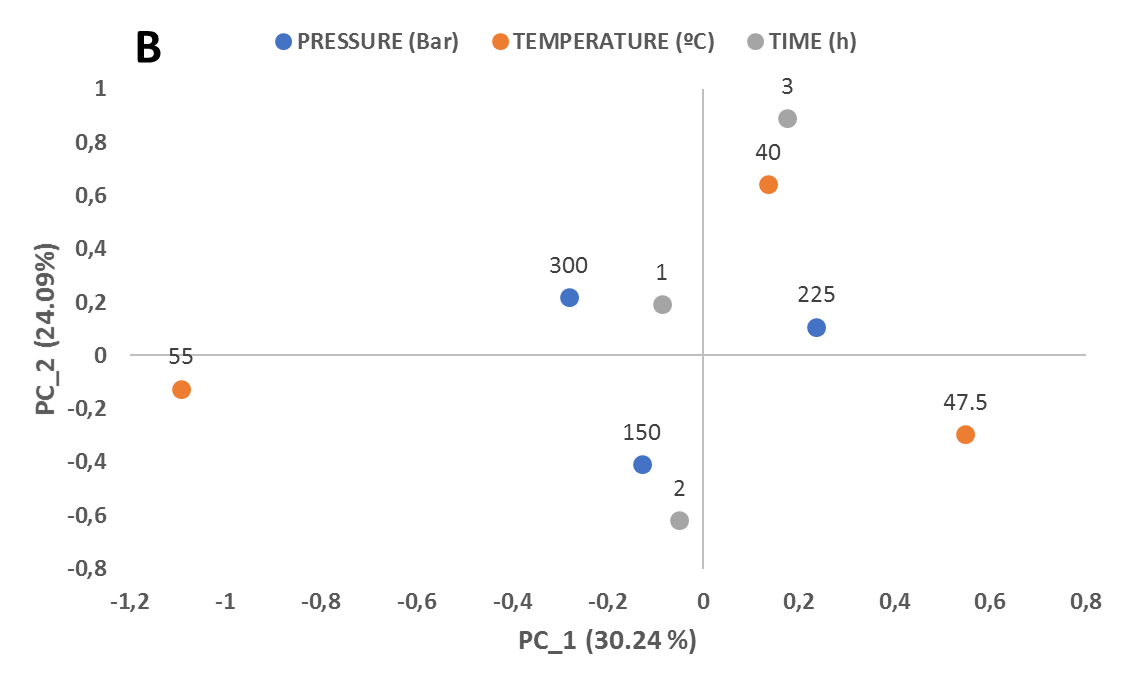


Figure S1. Loading plot (A) and score plot (B) after principal component analysis of components and functional properties of dietary fibre treated with supercritical fluids in the planes defined by the two first principal components (PC_1 and PC_2). Ws: solubility; Sw: swelling; WRC: water retention capacity; FAC: fat adsorption capacity; GAC: glucose absorption capacity; FNE: total non-extractable phenolic compounds; DPPH and ABTS: antioxidant activity of extract; Oligos: oligosaccharide degree of polymerization between 7-12; Agal: Galacturonic acid; Glu: Glucose; Xyl: Xylose; Ara: Arabinose; Fuc: Fucose.
